# Simulation of Reactive Fluoropolymer-Based Material Penetrator

**DOI:** 10.3390/ma17235822

**Published:** 2024-11-27

**Authors:** Peiyu Li, Zhenyang Liu, Jiahao Zhang, Mengmeng Guo, Qingbo Yu

**Affiliations:** China State Key Laboratory of Explosion Science and Technology, Beijing Institute of Technology, Beijing 100081, China; 3120225098@bit.edu.cn (P.L.); 3120215137@bit.edu.cn (Z.L.); 3120215138@bit.edu.cn (J.Z.); 3120195168@bit.edu.cn (M.G.)

**Keywords:** fluoropolymer-based reactive materials, simulation methods, reactive penetrators

## Abstract

It is very important to solve the numerical simulation of fluoropolymer-based reactive materials in the process of engineering design. Although custom development techniques are rapidly being applied to numerical simulation problems of reactive materials, they are inconvenient for engineering designers to implement. This paper presents several simulation methods for fluoropolymer-based reactive materials that can be implemented on commercial software platforms. Comparative analyses were conducted on the intrusion–explosion simulation based on a segmented simulation, a simulation based on the Lee–Tarver EOS, and a simulation of impact response models based on the MPM-SICR algorithm. Additionally, the similarities between these methods and experiments were compared. The results show that the impact response model simulation method based on the MPM-SICR algorithm has certain advantages in describing the impact detonation characteristics of reactive materials. The research findings can provide design assistance and a reference for the application design and damage assessment of fluoropolymer-based reactive material penetrators.

## 1. Introduction

A small-caliber penetrating projectile is a type of ammunition that penetrates a target with kinetic energy. Inert metal penetrating projectiles mainly damage targets through a penetrate-only approach, meaning that after piercing the target with kinetic energy, the material mainly relies on residual kinetic energy and fragmentation to inflict damage. In contrast, reactive materials primarily damage targets via penetration and then detonation. This means that after penetrating the target with kinetic energy, a portion of the reactive material is activated to undergo a chemical reaction, while the remaining intact material continues to penetrate the target via kinetic energy. Through the combined action of kinetic energy and chemical energy, damage to the target behind the target is achieved [1].

Fluoropolymer-based reactive materials are currently widely used [2]. However, due to differences in impact initiation and reaction characteristics when compared to explosives, the rules and numerical simulations for the impact initiation of fluoropolymer-based reactive materials remain in a developmental stage.

Tian [3] established a heterogeneous chemical reaction kinetic model for calculating the behavior of chemical reactions induced by impact in reactive materials. This model considers issues such as the increase in the temperature of reactants upon the initial impact, the reaction growth rate, and the mass transfer between interfaces. This model describes the reaction behavior and energy release characteristics of reactive materials. The rationality of the theoretical model was validated via impact energy release experiments in quasi-closed reaction vessels. Zhou [4] developed a chemical reaction model of fluoropolymer-based reactive materials and first proposed the idea of using a gas–solid two-phase reaction model to describe the reaction process of these materials. The shock initiation and reaction characteristics of reactive materials have also been studied [5,6,7,8,9,10]. However, these studies did not involve numerical calculations, and so the analyses have remained purely theoretical.

Lu [11] established impact ignition and gas–solid chemical reaction kinetic models for numerical calculations of reactive materials. Their models can accurately calculate impact initiation in reactive material, but embedding the model in AUTODYN18.0 software results in a low calculation efficiency. Xiao [12,13] utilized stress/temperature ignition criteria and Arrhenius reaction kinetic models to calculate the impact initiation in reactive materials and embed the numerical model into AUTODYN software. The model was embedded into MPM3D3.0 software to increase the computational efficiency.

Li [14] obtained EOS JWL parameters via cylinder experiments. Jiang [15] studied EOS JWL parameters and applied them in simulations. Rosencrantz [16] studied the Lee–Tarver EOS parameters of reactive materials, obtaining results via simulation. Some studies [17,18] have used these parameters to study the damage caused by reactive penetrators through numerical simulations.

Multilayer impact-resistant structures have been widely studied [19,20]. Reactive materials have been applied in striking multilayer structures owing to their characteristics. To address the inconvenience faced by engineers in implementing secondary development techniques, we developed three methods of simulating reactive projectiles using AUTODYN (ANSYS18.0 U.S.) and MPM3D3.0 which were developed and are freely available from Tsinghua Computational Dynamics Lab. The differences between the simulation results and the numerical model were compared, providing a reference for relevant design engineers.

## 2. The Experimental Effects of Reactive Penetrators

The impact of reactive material projectiles on multilayer spaced targets was chosen as the study object, with the aim of analyzing the effects of reactive materials after target damage. The experimental results served as the reference standard for our simulations.

### 2.1. Reactive Material Composite Liner

**Figure 1:**
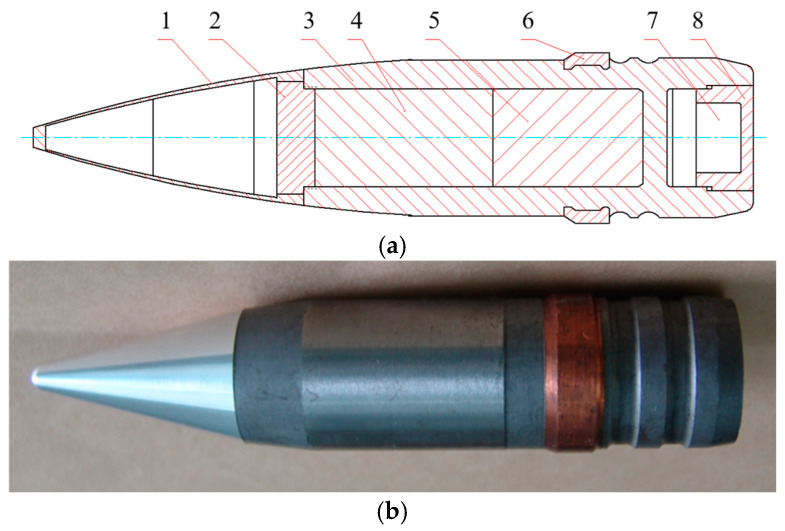
Structure of a projectile. (**a**) Bullet section diagram. (**b**) Internal components and appearance of the warhead. 1—Hood, 2—tungsten alloy block, 3—shell, 4—reactive core, 5—reactive core, 6—bearing band, 7—wheel balance weight, and 8—bottom screw.

In the experiment, the projectile core comprised pressed and sintered hardened PTFE/Al (PTFE 73.5 wt%; Al 26.5 wt%) energetic material with dimensions of Φ20 mm × 70 mm. A tungsten block was placed in front of the reactive core to enhance the penetration capability of the projectile. The dimensions of the projectile were Φ30 mm × 180 mm. The structure and exterior of the projectile are illustrated in Figure 1.

**Figure 2:**
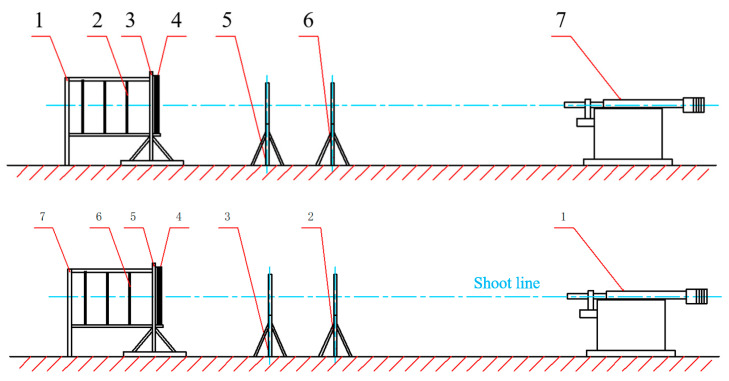
The experimental setup. 1. effect target frame, 2. effect target, 3. target frame, 4. steel target, 5. velocity measurement system, 6. velocity measurement system, 7. the 30 mm ballistic gun.

A ballistic impact experiment was conducted to study the post-penetration damage effects of projectiles with a reactive material core penetrating multilayer spaced targets. The setup of the ballistic impact experiment is illustrated in Figure 2. The experimental system primarily consisted of a 30 mm caliber ballistic gun, an RHA steel target (15 mm), a 2024-T3 aluminum target (5 mm), a 2 × Q235 steel target (1.2 mm), and velocity measurement with a high-speed camera. The distance between the muzzle of the ballistic gun and the target was 15 m.

### 2.2. Experimental Results

**Figure 3:**
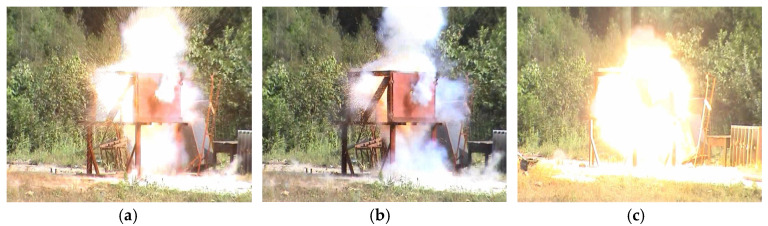
High speed photography of a projectile penetrating a target. (**a**) Penetrating the first layer of target plate. (**b**) Pass through the first layer of target plate. (**c**) Impact the target plate of the second layer.

The mechanism by which reactive materials penetrate a material differs from that of traditional inert metal fragments. Reactive materials are activated to undergo exothermic reactions upon target impact and penetration. The released chemical energy acts on the target plate during penetration. Penetration processes induce an exothermic reaction. Following the penetration of the armored steel plate, unreacted materials and projectile fragments continue to penetrate subsequent target plates, triggering further exothermic reactions. The overall process is depicted in Figure 3.

**Figure 4:**
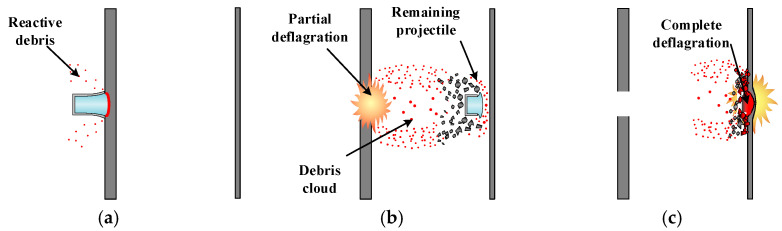
Impact behaviors of reactive materials on multi-layer targets. (**a**) Impact; (**b**) Partial deflagrate; (**c**) Secondary impact detonation.

Under intense dynamic loading during the collision process, reactive materials undergo activation and exothermic chemical reactions, producing a coupled response of kinetic energy and chemical energy. Using high-speed photography, the damage behavior of reactive materials impacting double-layer spaced plates can be abstractly divided into three stages, as shown in Figure 4.

## 3. Numerical Simulation of Reactive Projectile Penetration

### 3.1. Simulation Based on the Segmented Simulation Method

We applied a previously described segmented simulation methodology [21] in our subsequent studies of reactive materials. An inert material model was used to simulate the penetration process and determine the internal pressure of the material. Subsequently, the ignition criterion for reactive materials proposed by Mock [22] was considered to determine whether the reactive material reacts. Following penetration, the material was replaced with a reactive material described by the JWL equation.

**Figure 5:**
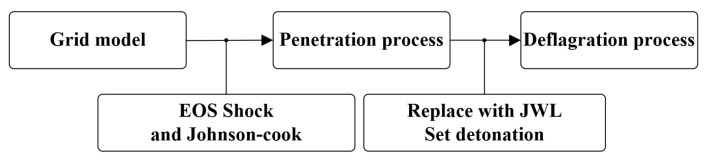
Segmentation simulation method.

The numerical simulation study was conducted using the ANSYS AUTODYN 3D platform. AUTODYN features various outstanding solvers, including Euler, Lagrange, ALE, and SPH, along with multiple material databases and algorithmic techniques. The nonlinear problems it tackles encompass large deformation and geometrical nonlinearity, plasticity, failure, strain hardening and softening, as well as material nonlinearity, which are described using segmented EOSs. Of the algorithms in AUTODYN, the Lagrange algorithm is primarily employed for simulating the nonlinear dynamics of solids and structures, while the smoothed particle hydrodynamics (SPH) algorithm is a meshless numerical technique that is particularly effective for handling mechanics problems involving high-speed collisions or brittle materials. The segmented simulation method is detailed in AUTODYN. An inert simulation was first performed to check whether detonation occurred; second, the inert material was replaced with the JWL equation; and a detonation point was manually added to ignite the JWL material. The main reason we used AUTODYN for the simulation is that AUTODYN allows the user to easily replace the material equations in the calculation process. The specific operational workflow is illustrated in Figure 5.

**Figure 6:**
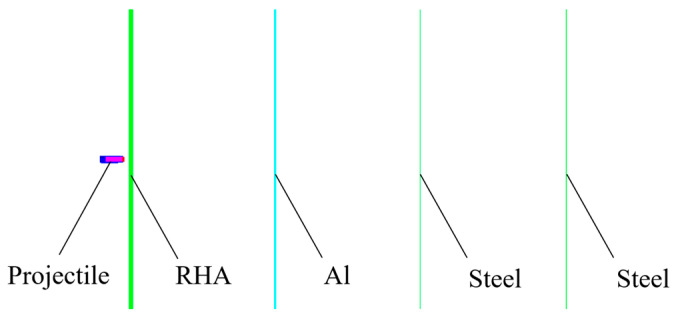
Grid model.

Considering the symmetry during the impact process, a one-quarter simulation of the penetration of a reactive core projectile through multiple layers of targets was established using grid generation software (True-Grid3.13) and the finite element simulation software (AUTODYN). The ogive of the reactive core projectile, composed of a thin layer of aluminum alloy, was neglected in the model. The tungsten head, shell, and core of the projectile were partitioned using SPH grids, whereas the target plate was partitioned using Lagrange grids. The target plate was subjected to fixed boundaries. The grid size influenced the accuracy of the calculations; therefore, the area where the target plate was impacted was meshed with a grid size of 2 mm × 2 mm × 2 mm, and the penetration body was meshed with a grid size of 1 mm × 1 mm × 1 mm. The computational model is depicted in Figure 6.

In the calculation process, the Johnson–Cook and EOS Grüneisen methods were used to describe the steel, tungsten alloy, aluminum, and nonreactive materials. The Johnson–Cook strength model is expressed as

(1) $$\sigma_{y}=\left(A+B\varepsilon_{p}^{N}\right)\left[1+C\ln\left(\frac{{\dot{\varepsilon }}_{p}}{{\dot{\varepsilon }}_{0}}\right)\right]\left[1-{\left(\frac{T-T_{0}}{T_{m}-T_{0}}\right)}^{m}\right]$$

where the three product terms represent the strain, strain rate, and temperature effects, respectively; *A* is the yield strength of the material; *B* denotes the strain hardening constant of the material; *N* denotes the strain hardening index; $\varepsilon_{p}$ denotes the effective plastic strain; *C* denotes the strain rate hardening coefficient; ${\dot{\varepsilon }}_{p}$ denotes the plastic strain rate; ${\dot{\varepsilon }}_{0}$ denotes the critical strain rate; *m* denotes the thermal softening coefficient; *T* denotes the initial temperature; *T*_m_ denotes the melting point of metal; and *T* denotes the material process temperature. The failure strain given in the material model is

(2) $$\varepsilon_{f}=\left[D_{1}+D_{2}e^{D_{3}\sigma^{*}}\right]\left[1+D_{4}\ln{\dot{\varepsilon }}^{*}\right]\left[1+D_{5}T^{*}\right]$$

where $\sigma^{*}$ denotes the ratio of pressure to effective stress; ${\dot{\varepsilon }}^{*}$ denotes the ratio of effective plastic strain rate to the quasi-static critical rate; $T^{*}$ denotes the specific temperature; and *D*_1_–*D*_5_ are corresponding constants.

EOS Grüneisen can be expressed as

(3) $$P=\frac{\rho_{0}c_{0}^{2}\eta }{\left(1-s\eta \right)}\left(1-\frac{\gamma_{0}\eta }{2}\right)+\gamma_{0}\rho_{0}E_{m}$$

where *P* denotes the pressure state of material;$\;\rho_{0}$ denotes the initial density; $c_{0}$ denotes the sound velocity of the material; *s* denotes the material coefficient; $\gamma_{0}$ denotes the Grüneisen coefficient; $E_{m}$ denotes the internal energy; and $\eta =1-\rho_{0}/\rho$.

In the simulation, steel, aluminum, and the tungsten alloy were represented by the above constitutive equations and EOS, with the specific parameters listed in Table 1, Table 2, Table 3 and Table 4.

For the detonated reactivated material, we used the JWL equation to describe the pressure state of the reaction products. The basic form of the JWL equation is

(4) $$P=A\left(1-\frac{w\eta }{R_{1}}\right)e^{-\frac{R_{1}}{\eta }}+B\left(1-\frac{w\eta }{R_{2}}\right)e^{-\frac{R_{2}}{\eta }}+w\rho E_{m}$$

where *P* denotes the pressure state of material;  ρ denotes the density of the material;$\;\eta =1-\rho_{0}/\rho$; $E_{m}$ denotes the internal energy; and *w*, *R*_1_, *R*_2_, *A*, and *B* are coefficients that can be obtained through cylindrical experiments [14]. The parameters’ values are listed in Table 5.

The activated portion of the reactive material was determined according to a previously described method [21]. Several observation points are set up along the axial direction of the reactive material core. During the impact of the projectile with the target plate, if the pressure peak at the observation points exceeds the critical activation pressure of the reactive material, the reactive material at that location is considered effectively activated during the impact process. The impact activation pressure is 300 MPa [21].

**Figure 7:**
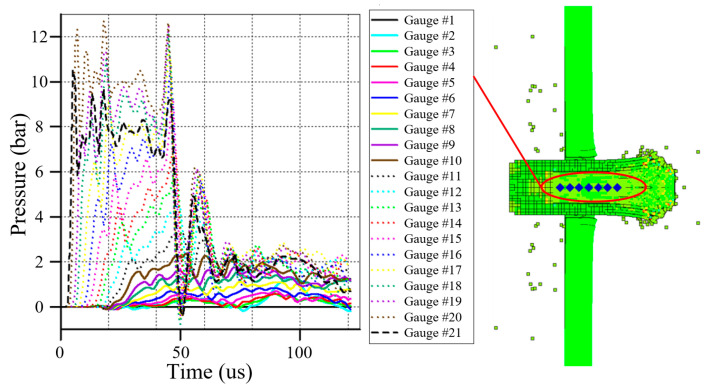
Pressure during target penetration.

We recorded and analyzed the pressure history at the observation point, finding that the historical pressure peak in almost all areas of the core exceeded 300 MPa. The main reason for the activation of all materials during penetration was attributed to the thick tar-get plate, resulting in slow pressure decay during penetration. Additionally, the steel shell constrained the core, resulting in a minimal influence from lateral rarefaction waves. These combined factors contributed to the nearly complete activation of the reactive material core during the penetration of the first layer of the target plate. Figure 7 depicts the pressure recorded at the observation point in the core during penetration, showing that the peak pressure of the core material near the bottom of the bullet was lower.

**Figure 8:**
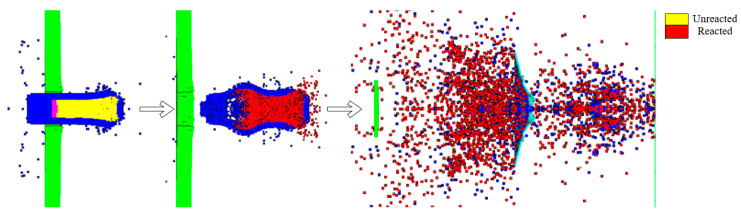
Penetration and explosion processes.

According to the simulation method outlined in Reference [21], after the reactive core penetrated the target plate, we changed the EOS for the parts of the core where the pressure exceeded 300 MPa. The JWL parameters in Table 5 were applied, and then the initiation points were set in the core. Figure 8 shows a typical penetration process using a simulation of an encroachment section.

**Figure 9:**
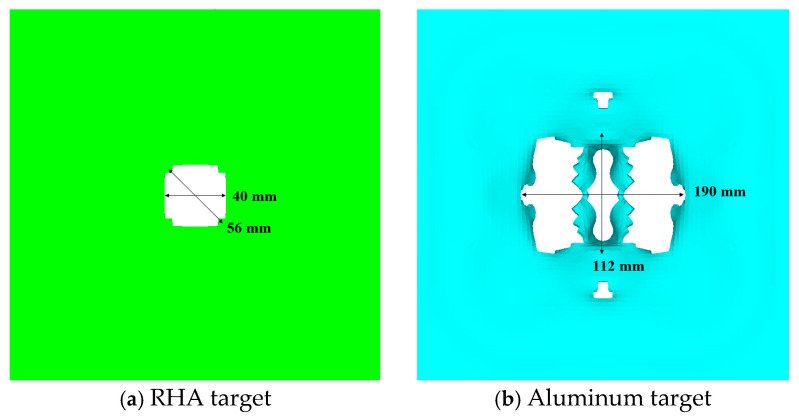

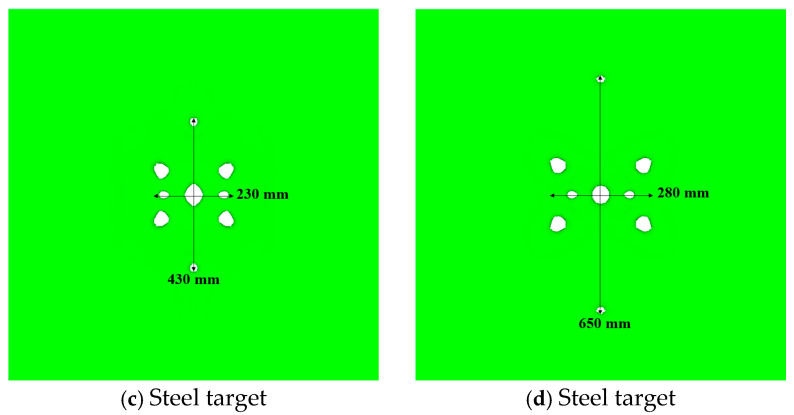
Results of projectile penetration.

The results of the interaction between the reactive penetrator, the steel target encountered, and the subsequent target plates are depicted in Figure 9. The steel target encountered by the reactive penetrator exhibited circular perforations nearing Φ 50 mm in diameter. The first 5 mm post-target aluminum plate was punctured with a hole measuring 190 mm × 112 mm due to the impact of the fragmentation cloud. Perforations in the second and third 1.2 mm post-target plates primarily stemmed from the residual large fragments of the shattered casing of the projectile. Additionally, owing to the expansion effect following the explosion of the reactive material, the radial velocity of the fragmented casing resulted in an expanding trend in the perforated area on the subsequent target plates.

### 3.2. Simulation Based on the Lee–Tarver EOS

Researchers [16] first introduced the use of the Lee–Tarver equation to describe the process through which reactive materials initiate under impact and determine the relevant parameters.

For the numerical simulation, we used the ANSYS AUTODYN 3D platform. Among the various algorithms in AUTODYN 3D, the Lagrange algorithm is primarily employed to simulate nonlinear dynamic problems of solids and structures, whereas the SPH algorithm is a meshless numerical technique that is particularly effective at handling high-/ultrahigh-speed collisions and brittle material mechanics problems. Specifically, we conducted partial optimizations, the results of which we compared to those of previously used methods [16,23], addressing certain compatibility issues with the simultaneous use of the Lee–Tarver and Johnson–Cook models in LS-DYNA.

The simultaneously use of the Lee–Tarver EOS and Johnson–Cook models in LS-DYNA prevents the program from performing the computation. The authors of [16] used the *MAT_NULL strength model to describe unreacted reactive materials, which was inappropriate and substantially impacted the calculation of penetration processes. In AUTODYN 3D, the Johnson–Cook strength model and the EOS Lee–Tarver model can be simultaneously employed. EOS Shock can better describe unreacted substances than the*MAT_NULL model.

The impact response process reactive material is described using the Lee–Tarver EOS, whereas the mechanical response of the unreacted portion is initially described using EOS Shock as follows:

(5) $$U=c_{0}+su_{p}$$

where *U, u*_p_, and $c_{0}$ denote the shock, particle, and acoustic velocities; and *s* is a parameter.

We used the JWL equation to describe the pressure state of the reaction products.

In the mixture of unreacted materials and reaction products, *F* defines the degree of reaction. The values of *F* range from zero to one, where zero indicates no reaction, and one indicates the complete reaction of the material. The basic form of reaction rate equation is

(6) $$\frac{\partial F}{\partial t}=FREQ\times {\left(1-F\right)}^{FRER}{\left(V_{e}^{-1}-1-CCRIT\right)}^{EETAL}+GROW1\times {\left(1-F\right)}^{ESI}F^{ARI}p^{EM}+GROW2\times {\left(1-F\right)}^{ES2}F^{AR2}p^{EN}$$

where FREQ, FRER, CCRIT, and EETAL are the material constants of the part where the ignition term is located; GROW1, ES1, AR1, and EM are the material constants of the part where the reaction growth term is located; and GROW2, ES1, AR1, and EN are the material constants of the part where the reaction completion term is located, all of which need to be calibrated through tests. The reactive material parameters are listed in Table 6 [16].

**Figure 10:**
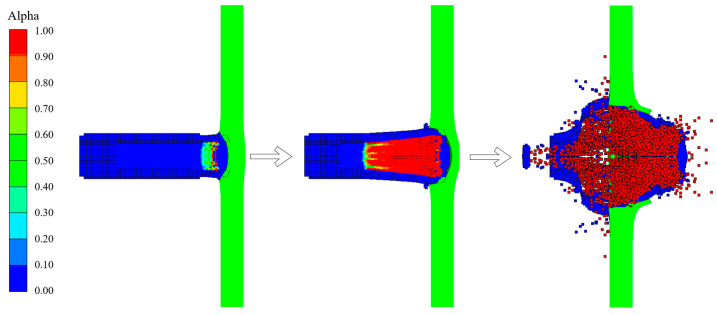
Impact detonation process.

The process of penetrating the steel target is illustrated in Figure 10. The core is subjected to the impact of shock waves, resulting in pressure and enabling spontaneous reactions to occur upon impact. During the reaction process, reactions occur as the pressure wave propagates. Compared with the segmented method, the reactions begin before penetrating the target plate, and considerable expansion of the shell occurs during penetration.

**Figure 11:**
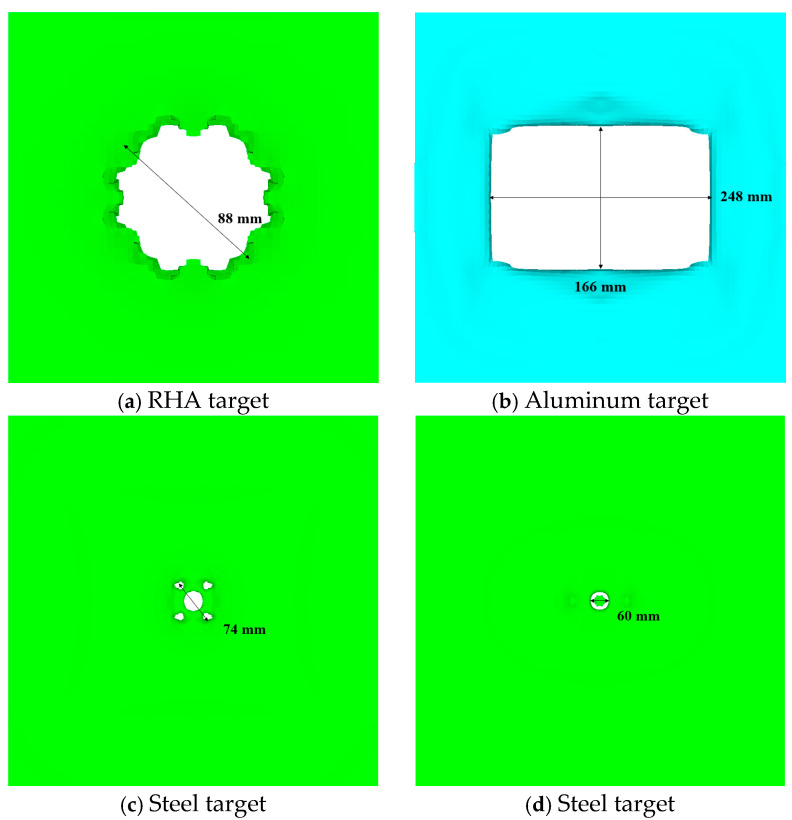
Results of projectile penetration.

The interaction between the penetrating projectile with a reactive material core and the steel and subsequent targets is shown in Figure 11. The steel target was perforated after being hit by the projectile, forming a circular through-hole nearing Φ88 mm in diameter The main reason for this is the strong radial expansion of the shell during the penetration process, which resulted in a strong expansion effect. The subsequent (first) 5 mm aluminum target was pierced by the impact of the fragment cloud, forming a rupture hole of 248 mm × 166 mm. The holes pierced in the next (second) 1.2 mm target plate were mainly caused by the remaining large fragments of the fractured projectile after penetration. No obvious holes formed owing to fragment penetration on the next (third) 1.2 mm target plate; the perforations in this target plate were mainly caused by the impact of the plug blocks from the previous target. Compared with the results of the segmented simulation method, fewer perforations were recorded in the last two target plates in our study. The main reason for this is that the reaction rate of the reactive material is higher and the initiation time was earlier, leading to larger diffusion of the fragment cloud. This means that the mass of the fragment cloud is more dispersed, resulting in smaller individual fragments and a decreased penetration capability, and causing few perforations in the last two target plates.

### 3.3. Simulation of the Impact Reaction Model Based on the MPM-SICR

Our numerical simulation mainly relied on MPM3.0, developed by Tsinghua University, for a platform. In MPM3.0, the Lagrange algorithm is mainly used to simulate the nonlinear dynamic problems associated with solids and structures; the MPM algorithm, on the other hand, is a meshless numerical technique used for material points, in which particles carry material state information. When updating the state, this information is mapped to the background grid, and stress–strain velocity updating processes are calculated using integral equations. The MPM algorithm is particularly effective at handling mechanics problems such as high-/ultrahigh-speed collisions and brittle materials. The state equation for reactive materials was derived from the literature [13], employing an improved impact initiation model based on the Lee–Tarver state equation, which was integrated into the MPM3.0 platform. The software platform was available for trial through the Tsinghua Computational Dynamics Lab.

**Figure 12:**
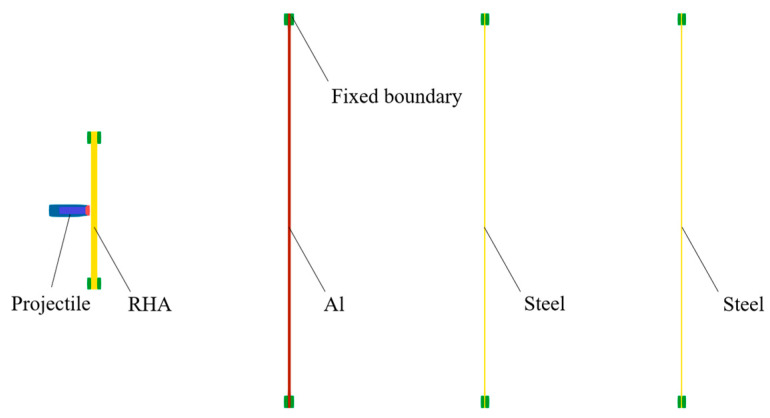
Grid model of MPM.

A one-quarter numerical simulation of the penetration of an active core projectile into a multilayer target was established using SolidWorks2020 modeling software and LS-PERPOST finite element simulation software. The wind cap of the active core projectile, composed of thin-layer aluminum alloy, was neglected in the modeling process, and all grids were implemented using the MPM method. The particle size used for the projectile was 1.2 mm’ for the target plate, and the particle size ranged from 1.2 mm to 2.5 mm. Due to the inability to set boundary conditions in the MPM3D program, two solid blocks with constant velocities of zero were used to sandwich the target plate. The partitioned grid model is illustrated in Figure 12.

**Figure 13:**
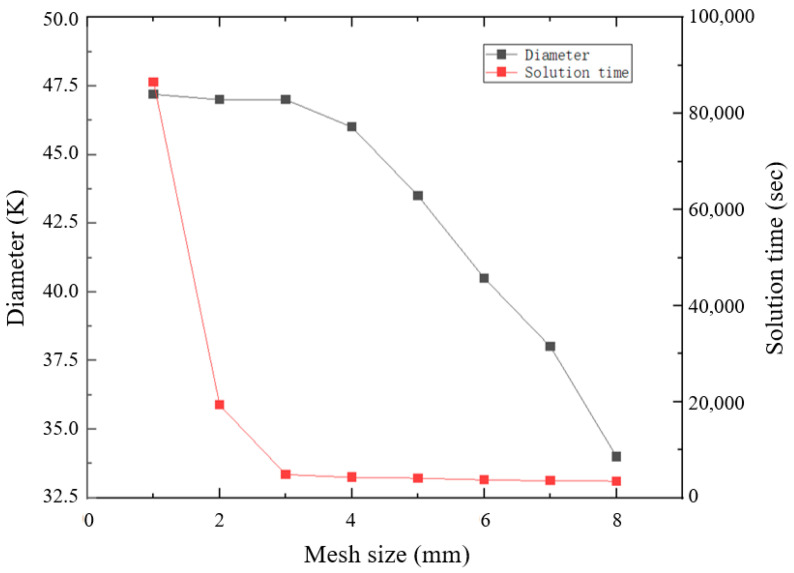
Mesh convergence of MPM.

We analyzed the grid convergence on MPM-SICR. We did not verify convergence for the first two methods because the literature provides suggestions for the grid size. Convergence was verified using the diameter of the fragmented cloud at a certain moment after penetrating the first layer of the target plate as a variable. The results of the analysis are shown in Figure 13. When the grid size was reduced to 3 mm, the diameter of the fragmented cloud no longer substantially changed. The time for obtaining a solution was notably increased when the grid was reduced to 3 mm.

During the simulation, for the core material, we used the impact initiation model of the reactive material, applying the parameters listed in Table 7 and Table 8.

**Figure 14:**
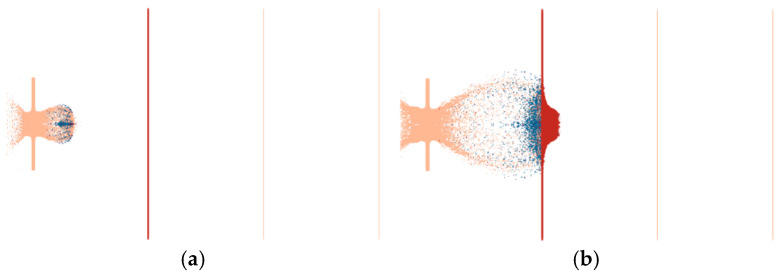
The penetration process obtained by the MPM method. (**a**) Penetrating the RHA plate at 240 us; (**b**) Penetrating the aluminum plate at 680 us.

The impact initiation equation of the reactive material in this study was a model dominated by the Arrhenius formula for the reaction rate, with pressure and temperature as reaction thresholds. The process of the core penetrating the target plate is illustrated in Figure 14, where the core is affected by the impact, reaching the reaction threshold and spontaneously reacting upon impact. The projectile begins to react before penetrating the target plate, and significant expansion occurs in the shell during penetration, leading to a strong spalling effect and forming a fragment cloud containing a mixture of the target plate, shell, and core material. Compared with the previous two simulation methods, the main characteristic of the proposed method is that during the penetration of the RHA, the core does not completely react, leaving a considerable amount of unreacted material. Moreover, the reaction continues during the subsequent penetration of the aluminum target plate. In terms of the process, the results of this approach are closer to those obtained via the experimental high-speed photography and the reaction mode proposed in Section 2.2.

**Figure 15:**
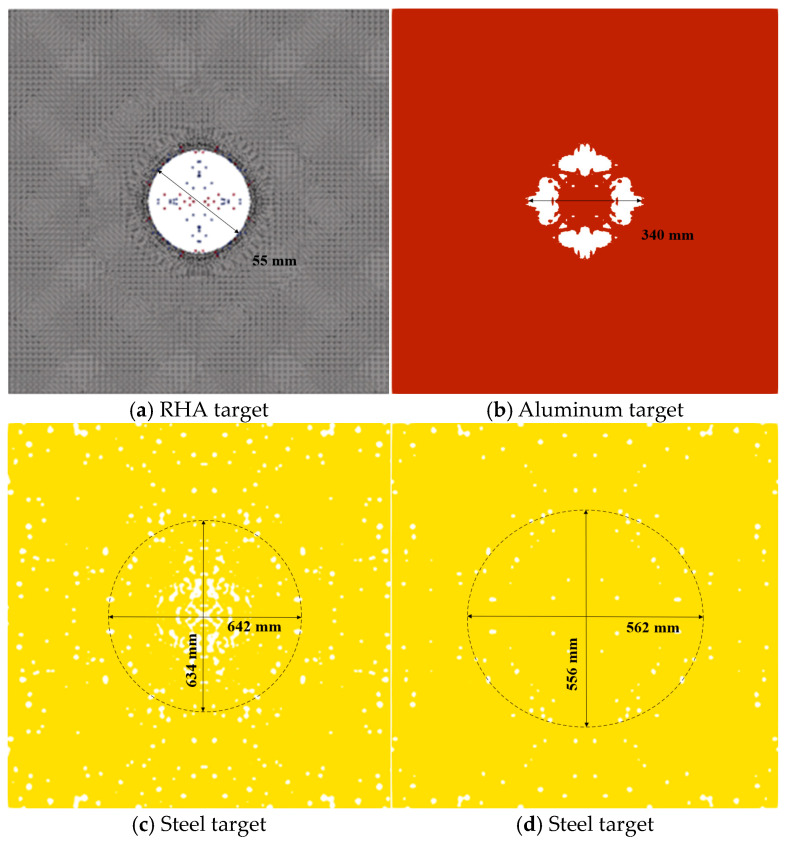
Results of projectile penetration.

The interaction between the projectile and the target plate is shown in Figure 15. After being struck by the projectile, the steel target plate had circular through-holes of approximately Φ 55 mm in diameter. The main reason for the circular through-holes that were larger than the projectile diameter was the radial expansion of the shell during penetration, which resulted in a spalling effect. The first 5 mm aluminum target plate was pierced by the impact of the fragment cloud, forming a Φ 340 mm rupture hole. The perforations in the second 1.2 mm target plate primarily resulted from the fractured shell of the penetrating projectile and fragments from the previous target. Similarly, fewer but visible perforations were caused by fragment penetration in the third 1.2 mm target plate. The main reasons for the lower number of perforations in the latter two target plates were the decreases in projectile velocity and penetration capability after the fragments penetrated the second target, resulting in a reduced ability to penetrate.

## 4. Comparison of Numerical Simulation Methods and Discussion

### 4.1. Fragment Scattering Morphology and Reactivity

**Figure 16:**
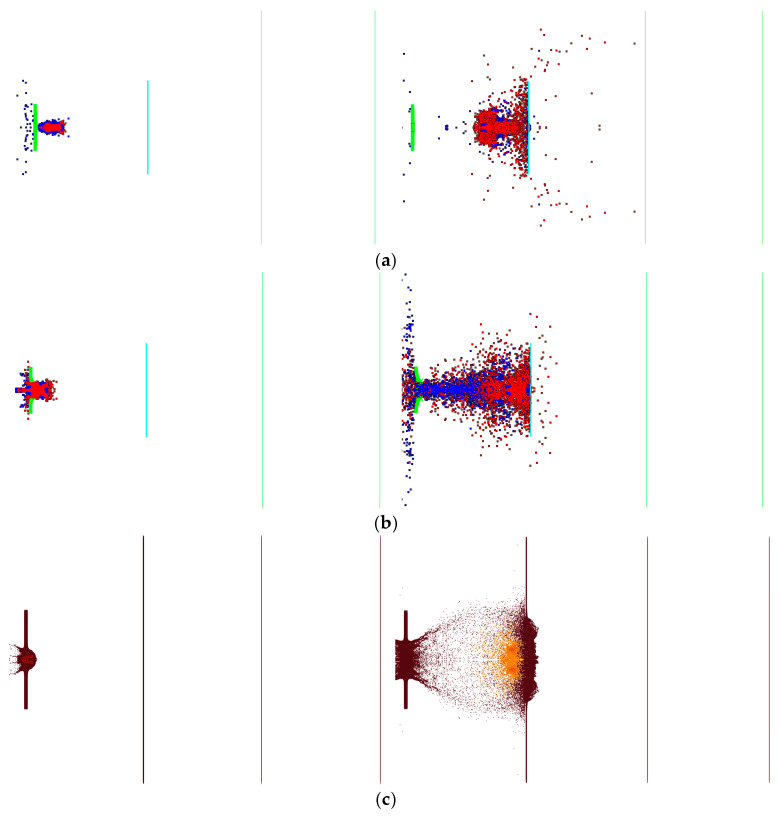
Comparison of the results for the fragment scattering morphology and reactivity. (**a**) Segmented simulation of intrusion and explosion. (**b**) Simulation based on the EOS Lee–Tarver method. (**c**) Simulation of the impact initiation model based on the MPM-SICR algorithm.

Figure 16 compares the three numerical simulation methods used to describe the target penetration process. The segmented penetration and explosion simulation method failed to capture the characteristics of the secondary collision and secondary explosion, which were observed via high-speed photography. The main issue was the need to replace the materials manually and decide on initiation during the simulation process, which reduced the stability and accuracy of the numerical simulation. Moreover, this method failed to reflect the non-self-sustaining reaction characteristic of reactive materials, where all reactions occur once initiated. The simulation based on the Lee–Tarver state equation also failed to capture the characteristics of the secondary collision and secondary explosion observed via high-speed photography because the parameters of the Lee–Tarver initiation equation are inherently designed to describe the reaction time scale of explosives. When applied to describe reactive materials, the reaction speed is too fast, and reactive materials exhibit pressure initiation threshold characteristics, which are not reflected by the Lee–Tarver equation. Both methods produce excessively rapid reaction accumulation, with all reactions occurring after penetrating the first layer of the target plate. The damage effect reflected on the target plate mainly manifests in an insufficient number of perforations in the third and fourth plates. The simulation with the MPM-SICR algorithm’s shock reaction model roughly reflected the experimental results regarding the damage process. The collision with an aluminum target plate exhibits characteristics similar to the secondary collision and explosion observed with high-speed photography, essentially reflecting the characteristics of the pressure initiation threshold of the reactive materials.

**Figure 17:**
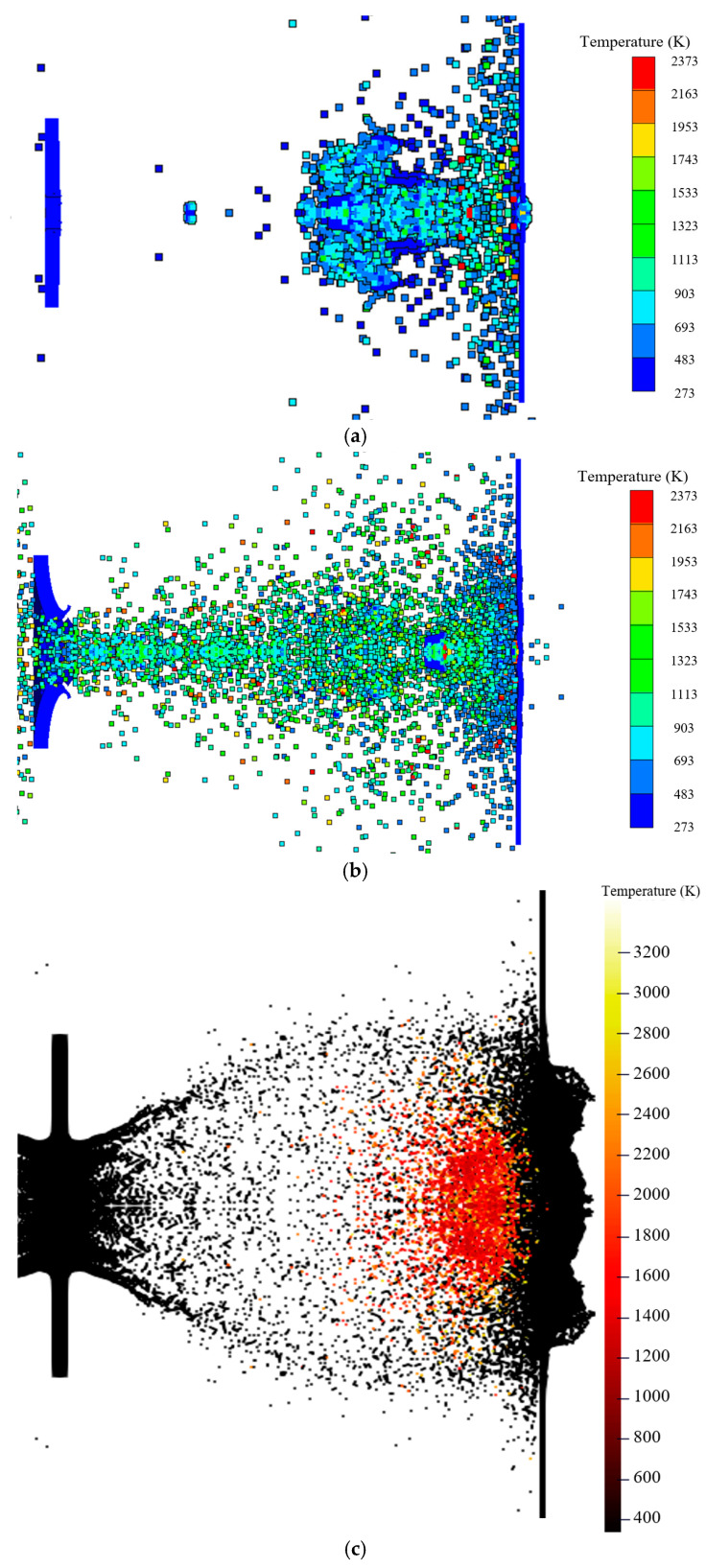
Comparison of the reaction temperatures using the three methods. (**a**) Segmented simulation of intrusion and explosion. (**b**) Simulation based on the EOS Lee–Tarver method. (**c**) Simulation of the impact initiation model based on the MPM-SICR algorithm.

Figure 17 compares the temperature fields during the penetration process obtained using the three numerical simulation methods. In the simulations based on segmented penetration, the Lee–Tarver state equation, and the MPM-SICR algorithm’s impact reaction model, most of the particle temperatures are approximately 900 K, 1300 K, and 2300 K, respectively.

### 4.2. Target Damage

**Figure 18:**
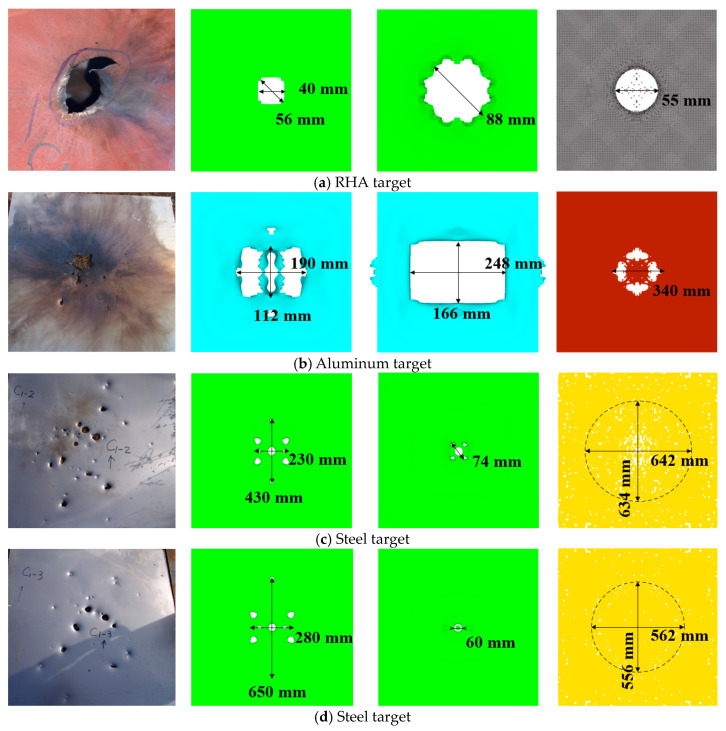
Comparison of the results for target damage.

We compared the characteristics of the three methods and the accuracy with which they described the experimental results. Figure 18, from left to right, displays the results of the comparative tests of the segmented penetration and explosion simulation, the simulation based on the EOS Lee–Tarver method, and the simulation with the impact reaction model based on the MPM-SICR algorithm. The four rows from top to bottom represent the four target plates.

### 4.3. Discussion of the Methods’ Characteristics

The segmented simulation approximately replicated the experimental results of the reactive penetrator in terms of target plate damage modes. However, the description of the damage process was somewhat inadequate: the characteristics of the secondary collision and secondary explosion observed with high-speed photography were not captured. The main issues with this simulation were the need to replace the materials and the requirement to manually set off the detonation, which compromised the stability and accuracy of the numerical simulations. Moreover, this method failed to reflect the unstained reaction characteristics of the reactive materials, as the entire reaction occurred upon initiation. Although the simulation results approximated the damage outcomes, the number of perforations in the subsequent target plates was low.

With the simulation using the Lee–Tarver state equation, we could approximately re-produce the experimental results obtained with the reactive core penetrating projectiles regarding the damage pattern on the target plate. However, this method failed to capture characteristics such as the secondary collision and secondary detonation observed with high-speed photography. This issue arose because the parameters of the Lee–Tarver detonation equation were originally designed to describe the reaction process of explosives, resulting in an excessively rapid reaction rate when applied to describe active materials. Regarding the simulated materials, active materials are characterized by a large pressure detonation threshold, which the Lee–Tarver equation fails to capture. Although the dam-age effects were approximately reproduced, the number of perforations in the subsequent target plate was low and the dispersion range was limited.

The simulation conducted using the impact reaction model with the MPM-SICR algorithm approximately reproduced the experimental results obtained with the reactive penetrators. The simulation results roughly depicted the damage process, capturing the experimental phenomena and characteristics such as the secondary collisions and detonations observed via high-speed photography. Furthermore, the simulation results reflected the pressure initiation threshold characteristics of the reactive material.

## 5. Conclusions

We introduced a segmented penetration and explosion simulation, a Lee–Tarver state equation-based simulation, and an impact reaction model simulation based on the MPM-SICR algorithm. The simulation results of these three methods were compared with the experimental results, leading to the following conclusions:

(a) The results of three methods of simulating reactive materials were compared: segmented penetration and explosion simulation, Lee–Tarver state equation-based simulation, and impact reaction model simulation based on the MPM-SICR algorithm. The impact reaction model simulation method based on the MPM-SICR algorithm displayed certain advantages in describing the characteristics of the detonation of a projectile with an active material core upon impact. This method could more accurately reflect the characteristics of multiple impact detonations and the detonation morphology that were observed in the experiments.
(b) The parameters related to reaction energy in all three models were derived from actual measurements of chemical reactions, and the calculation models did not include air. Therefore, the resulting reaction temperatures calculated using the models were not substantially different.
(c) After comparing the damage effects simulated using the three models, the results obtained with the impact reaction model based on the MPM-SICR algorithm more accurately simulated the perforation morphology of the target plate. However, excessive fragility was observed in the subsequent target plates due to the poor simulation effect of the MPM algorithm for thin shells. Although the damage morphology was similar to that observed in the experiment, the damaged area differed from that in the experiment.

## Figures and Tables

**Table 1:** Parameters of steel.

***ρ*/(kg·m^−3^)**	***E*/GPa**	***C*_0_/(m·s^−1^)**	***S***	***γ***	***H***
7800	210	4580	1.490	2.17	1
***Tm*/K**	***A*/MPa**	***B*/MPa**	***n***	***C***	***m***
1795	1506	320	0.12	0.016	1.06
$\dot{\varepsilon }$ **/s^−1^**	***D*_1_**	***D*_2_**	***D*_3_**	***D*_4_**	***D*_5_**
1	0.01	2.73	1.57	2.00	0.61

**Table 2:** Parameters of RHA.

***ρ*/(kg·m^−3^)**	***E*/GPa**	***C*_0_/(m·s^−1^)**	***S***	***γ***	***H***
7800	200	4569	1.490	2.17	1
***Tm*/K**	***A*/MPa**	***B*/MPa**	***n***	***C***	***m***
1795	792	320	0.28	0.064	1.06
$\dot{\varepsilon }$ **/s−1**	***D*_1_**	***D*_2_**	***D*_3_**	***D*_4_**	***D*_5_**
1	0.1	0.76	1.57	0.005	−0.84

**Table 3:** Parameters of the tungsten alloy.

***ρ*/(kg·m^−3^)**	***G*/GPa**	***C*_0_/(m·s^−1^)**	***S***	***γ***	$\dot{\varepsilon }$ **/s^−1^**
17,000	160	4029	1.237	1.54	1
***Tm*/K**	***A*/MPa**	***B*/MPa**	***n***	***C***	***m***
1723	1506	117	0.12	0.016	1

**Table 4:** Parameters of Al−2024.

***ρ*/(kg·m^−3^)**	***G*/GPa**	***C*_0_/(m·s^−1^)**	***S***	***γ***	$\dot{\varepsilon }$ **/s^−1^**
2785	27.6	5328	1.338	2.00	1
***Tm*/K**	***A*/MPa**	***B*/MPa**	***n***	***C***	***m***
775	265	426	0.34	0.015	1

**Table 5:** JWL parameters of the explosive.

*A*/Mbar	*B*/Mbar	*R*1	*R*2	*w*	*D_CJ_*/cm·us^−1^	*E_CJ_*/(Gerg·mm^−3^)
4.735 × 10^5^	30	50.47	4.9	0.314	0.12	0.32

**Table 6:** Parameters of the EOS Lee–Tarver model.

Parameter	Value	Parameter	Value	Parameter	Value	Parameter	Value
a	4.7355 × 10^5^	b	30.6203	xp_1_	50.466801	xp_2_	4.92737
FRER	1.7271	g	4.575 × 10^−6^	r_1_	2.293712	r_2_	2.009256
r_3_	7.7099 × 10^−5^	r_5_	45.292294	r_6_	51.545380	FMXIG	0
FREQ	11224	GROW1	5.452 × 10^9^	EM	16.735001	AR1	0
ES1	1.0029	CVP	1.457 × 10^−5^	CVR	9.93 × 10^−6^	EETAL	28.399
CCRIT	1 × 10^−6^	ENQ	0.0135	TMP0	0	GROW2	8.599 × 10^13^
AR2	6.3067 × 10^−5^	ES2	1.0002	EN	26.963		

**Table 7:** Material parameters of the Johnson–Cook and shock models.

***ρ*_0_ (g/cm^3^)**	***c_p_* (Terg/gK)**	***G* (Mbar)**	***A* (Mbar)**	***B* (Mbar)**	**C**	**n**
2.76	1.16 × 10^−5^	0.00667	0.0012	0.0105	0.4	1.8
***θ*_m_ (K)**	***θ*_r_ (K)**	***β***	***c*_0_ (m/s)**	**s**	**γ**	
500	294	1.0	1350	2.258	0.9	

**Table 8:** Impact detonation parameters.

	Unreacted Materials	Values		Reaction Products	Values
EOS Shock	*ρ*_0_/(g·cm^−3^)	2.27	EOS JWL	A/MPa	7.9724 × 10^5^
	Γ	0.9		B/MPa	22,576
	*c*_0_/(cm·μs^−3^)	0.14		R1	7
	s	9.25		R2	2
	*C*_v,u_/(J·g^−1^K^−1^)	1.2		ω	0.5
Ignition criterion	σ_TS_/MPa	300		*C*_v,p_/(J·g^−1^K^−1^)	1.0
	c	1	Lee–Tarver	E/GPa	19.36
	T_d_/K	700		freg/us^−1^	2.2
Johnson–Cook	G/MPa	666		ccrit	0.01
	A/MPa	8.044		frer	0.667
	B/MPa	250.6		eeta1	2
	N	1.8		grow1	20.7
	C	0.4		es1	0.222
	m	1		ar1	0.667
Johnson–Cook failure model	D1	0.34		em	1
	D2	0.0489		grow2	30.7
	D3	−3.03		es2	0.222
	D4	0.016		ar2	0.667
	D5	1.12		en	1

## Data Availability

Data will be made available upon request.
